# Cleaning of LTCC, PEN, and PCB Au electrodes towards reliable electrochemical measurements

**DOI:** 10.1038/s41598-022-23395-3

**Published:** 2022-11-28

**Authors:** Mahan Hosseinzadeh Fakhr, Natalia Beshchasna, Sascha Balakin, Ivan Lopez Carrasco, Alexander Heitbrink, Fabian Göhler, Niels Rösch, Joerg Opitz

**Affiliations:** 1 Fraunhofer Institute for Ceramic Technologies and Systems IKTS, 01109 Dresden, Germany; 2 Max Bergmann Center of Biomaterials (MBC), Technical University of Dresden, 01069 Dresden, Germany; 3 Institute of Physics, Chemnitz University of Technology, 09126 Chemnitz, Germany; 4 InnoME GmbH, 32339 Espelkamp, Germany

**Keywords:** Chemistry, Materials science, Physics

## Abstract

Surface cleaning of the working electrode has a key role in improved electrochemical and physicochemical properties of the biosensors. Herein, chemical oxidation in piranha, chemical cleaning in potassium hydroxide-hydrogen peroxide, combined (electro-) chemical alkaline treatment, and potential cycling in sulfuric acid were applied to gold finish electrode surfaces deposited onto three different substrates; low temperature co-fired ceramics (LTCC), polyethylene naphthalate (PEN), and polyimide (PI), using three different deposition technologies; screen printing, inkjet printing, and electroplating (printed circuit board technology, PCB) accordingly. The effects of the (electro-) chemical treatments on the gold content and electrochemical responses of LTCC, PEN, and PCB applicable for aptamer-based sensors are discussed. In order to assess the gold surface and to compare the efficiency of the respective cleaning procedures; cyclic voltammetry (CV), electrochemical impedance spectroscopy (EIS), X-ray photoelectron spectroscopy (XPS), and scanning electron microscopy (SEM) were employed. LTCC sensors electrochemically cycled in sulfuric acid resulted in the most gold content on the electrode surface, the lowest peak potential difference, and the highest charge transfer ability. While, for PEN, the highest elemental gold and the lowest peak-to-peak separation were achieved by a combined (electro-) chemical alkaline treatment. Gold content and electrochemical characteristics on the PCB surface with extremely thin gold layer could be slightly optimized with the chemical cleaning in KOH + H_2_O_2_. The proposed cleaning procedures might be generally applied to various kinds of Au electrodes fabricated with the same conditions comparable with those are introduced in this study.

## Introduction

Gold has been widely used as a transducer element in the field of electrochemical biosensor owing to its fascinating properties such as excellent electrical conductivity, chemical inertness, and superior biocompatibility^1–3^. Furthermore, a strong interaction between gold and thiol groups has attracted great attention towards the development of thiol-functionalized aptamers in biosensor application. Aptamers can be easily modified chemically to thiol-terminated molecules and immobilized on the gold surfaces via self-assembled monolayers (SAMs) of thiol-gold^4,5^. All these prominent features have highlighted the importance of Au electrodes and surface pretreatments prior to the immobilization and coupled with electrochemical sensing methods paved the way for development of a powerful tool for point-of-care (POC) biological diagnosis^6^. Not only the quantity and quality of thiol–gold interactions on the surface^7,8^, but also electrochemical detection performance^9,10^ are strongly affected by the surface cleanliness.

Commercially fabricated Au electrodes are subjected to adsorb a wide range of adventitious contaminants from the laboratory environment or during the manufacturing process. Contaminants on the gold surface can act as a barrier that block the covalent bonding between gold and sulfur group in thiolated aptamers, in addition to a resistance against charge transfer ability on the electrode surface; hence cleaning of the transducer substrate has been considered as the first and foremost step in fabrication of a highly sensitive electrochemical aptasensor and achieving reproducible results^11^.

Numerous cleaning approaches including chemical and electrochemical pretreatments for preparing reproducible gold surfaces have been proposed in previous works^9,11–13^ and the results were characterized using electrochemical and physicochemical methods^11–14^. UV-Ozone cleaning procedure has been known as a simple, dry, and effective method to remove a wide variety of organic impurities from various substrates. UV irradiation oxidizes the organic molecules either via O_3_ formation or direct dissociation of C–C and C–H bonds in the adsorbed organic compounds which can be much easier oxidized and removed from the surface^15–17^. Although piranha solution has been considered the most popular oxidizing agent to remove different kinds of impurities and organic materials from the gold surface^18–21^ some evidence of delamination of gold film from the substrate and topography damage have been reported^22,23^ in addition to the gold-oxide formation on the surface^23,24^ that requires to be removed with further cleaning procedures. Cyclic voltammetry (CV) in sulfuric acid solution is a commonly used method for gold surface cleaning^10,25,26^ to effectively polish the gold surfaces without changing the morphology^27^. Cleaning in alkaline (KOH + H_2_O_2_) solution to remove the organic impurities has also been reported elsewhere^28–30^. According to the literature, a potential sweep in KOH followed by chemical alkaline treatment (KOH + H_2_O_2_) is found to be the most effective method to leave a very clean gold surface while maintaining the electrochemical properties of the electrode^11,12,31^.

In this study, the most efficient cleaning procedure for LTCC, PEN, and PCB (-based) gold electrodes in terms of electrochemical responses and surface properties is explored. Various (electro-) chemical cleaning approaches have been tested on three types of Au electrodes fabricated with different technologies and processes to achieve the most electroactive surface area and the highest concentration of elemental gold. To this end, electroactivity and surface cleanliness were characterized using electrochemical methods: Cyclic voltammetry (CV) and electrochemical impedance spectroscopy (EIS), as well as physicochemical analysis: X-ray photoelectron spectroscopy (XPS) and Scanning electron microcopy (SEM).

## Experimental

### Materials

Sulfuric acid (98% H_2_SO_4_) and potassium hydroxide (KOH) were purchased from Sigma-Aldrich (https://www.sigmaaldrich.com/, St. Louis, USA) and Merck (https://www.merckmillipore.com/, Darmstadt, Germany) respectively. Hydrogen peroxide (30% H_2_O_2_), phosphate-buffered saline (PBS), potassium ferricyanide K_3_[Fe(CN)_6_], and potassium ferrocyanide K_4_[Fe(CN)_6_] were purchased from Carl Roth (https://www.carlroth.com/, Karlsruhe, Germany). All solutions were prepared in ultra-pure water (18.2 MΩ.cm at 25 °C) produced by a Direct- UV Water Purification System purchased from Merck (https://www.merckmillipore.com/, Darmstadt, Germany) and freshly utilized.

### Working electrode

Three types of Au electrodes fabricated with different printing technologies on different substrate were employed as the working electrode in this study as follows: First, LTCC (Low Temperature Co-fired Ceramic) printed gold paste on GT951-ceramic substrate (30 mm by 8 mm with 0.07 cm^2^ surface area) utilizing thick-film technology. Second, PEN (Polyethylene Naphthalate) printed gold layer with 1 µm thickness on Polyethylene naphthalate polymer film (34 mm by 10 mm with 0.28 cm^2^ surface area) using inkjet printing technology. Both LTCC and PEN (-based) electrodes were manufactured in Fraunhofer Institute for Ceramic Technologies and Systems IKTS (https://www.ikts.fraunhofer.de/, Dresden, Germany), while PCB-based finish gold chips were fabricated via electroplating of the nickel-gold (Ni ≥ 1.5 µm, Au ≥ 0.03 µm) layer on a polyimide (PI) substrate containing copper as a printed circuit board (34 mm by 10 mm with 0.28 cm^2^ surface area) supplied by LeitOn GmbH (https://www.leiton.de/, 12105 Berlin, Germany).

### Cleaning procedure

**Figure 1:**
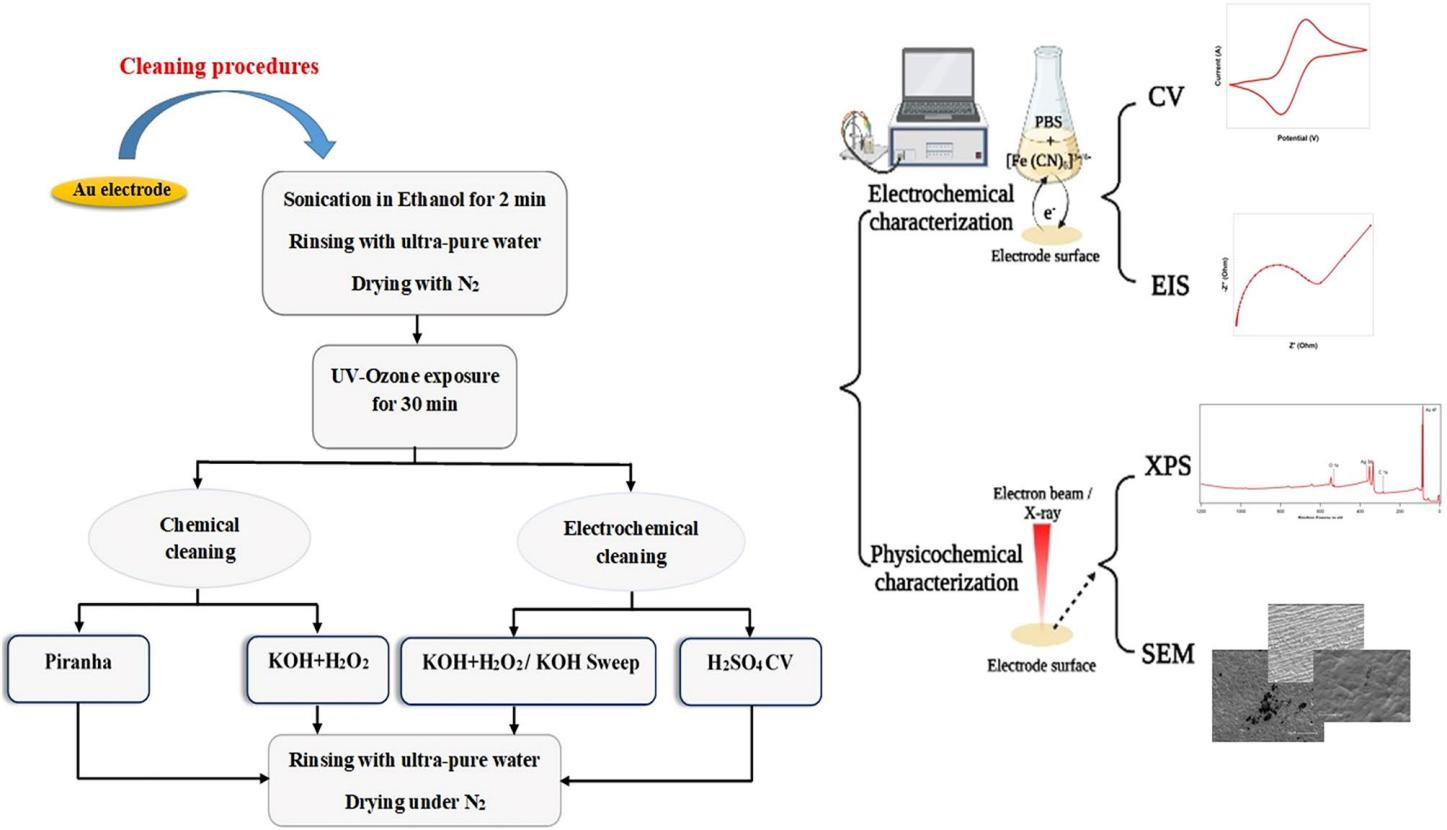
Schematic illustration of the cleaning procedures for different sensor electrodes and characterization methods.

Initially, Au electrodes were cleaned using ultrasound in ethanol for two minutes, rinsed thoroughly with ultra-pure water, and dried under nitrogen gas. To remove the organic contaminants, Au chips were exposed to UV irradiation in a UV Ozone Cleaner—ProCleaner purchased from BioForce Nanosciences (https://bioforcenano.com/, Chicago, USA) for 30 min. Shortened “UV-O_3_” in the results. Thereafter, UV-O_3_ cleaned sensors were cleaned chemically via chemical oxidation either in piranha or KOH + H_2_O_2_, and electrochemically via either a single potential sweep in KOH or potential cycling in H_2_SO_4_ following the UV-O_3_ pretreatment as shown in Fig. 1: Chemical oxidation in piranha, UV-O_3_ cleaned sensors were dipped in piranha solution (Three parts concentrated 98% H_2_SO_4_ and one part 30% H_2_O_2_) for 5 min (CAUTION: extreme precaution is needed on handling and using piranha solution!). Potassium hydroxide and hydrogen peroxide cleaning, UV-O_3_ treated chips were immersed in a solution (50 mM KOH and 30% H_2_O_2_ in the ratio of 3:1) for 10 min. Shortened “KOH + H_2_O_2_” in the results. Potassium hydroxide and hydrogen peroxide/KOH Sweep, Gold chips were sequentially cleaned in UV-Ozone photoreactor, KOH + H_2_O_2_ solution, and rinsed with ultra-pure water. A single linear potential was swept between − 200 and 1200 mV [vs. Ag/AgCl (sat. 4 M KCl)] in 50 mM KOH at a sweep rate of 50 mV/s. Shortened “KOH + H_2_O_2_ /KOH Sweep” in the results and also known as combined (electro-) chemical alkaline treatment. Potential cycling in sulfuric acid, UV-O_3_ cleaned chips were placed in a three-electrode electrochemical cell and the potential was cycled between − 500 and 1700 mV [vs. Ag/AgCl (sat. 4 M KCl)] at 100 mV/s scan rate in 0.5 M H_2_SO_4_ until a reproducible cyclic voltammogram of gold was achieved. The sulfuric acid solution was already deoxygenated using nitrogen flushing into the solution for 5 min prior to the experiments. The procedure is known as “H_2_SO_4_ CV” in the results. After each cleaning procedure, gold sensors were rinsed thoroughly with ultra-pure water, dried under N_2_, kept in the vacuum, and analyzed over the next day at the latest time. Each cleaning protocol was tested over multiple samples and studied individually for statistical analysis. All experiments and measurements were carried out at 25 °C.

### Apparatus

An IviumStat.h purchased from Ivium Technologies B.V. (https://www.ivium.com/, Eindhoven, Netherlands) was used to perform electrochemical measurements as well as electrochemical cleaning using a three-compartment electrochemical cell consisted of a Au electrode as the working electrode, a platinum rod as the auxiliary electrode, and Ag/AgCl reference electrode in saturated 4 M KCl solution at room temperature. Before and after treatment following suggested cleaning procedures, each Au chip was characterized with CV (current vs. potential) by sweeping the potential from − 100 to + 600 mV [vs. Ag/AgCl (sat. 4 M KCl)] at 20 mV/s scan rate in PBS solution (pH 7.4) containing 1 mM [Fe (CN)_6_]^3−/4−^ as a redox agent. Impedance values were recorded at an amplitude of 10 mV on a DC potential of 0.2 V, 60 data points logarithmically distributed within the frequency range of 0.1–100 kHz and fitted to the Randles equivalent circuit model^14^ using IviumSoft 4.1066 (https://www.ivium.com/software/). XPS measurements were conducted using a SPECS system developed by SPECSGROUP (https://www.specs-group.com/, Berlin, Germany) consisting of a SPECS XR 50 M X-ray source combined with a SPECS Focus 500 crystal monochromator, which provides Al K-alpha radiation, and a SPECS PHOIBOS 150 MCD-9 hemispherical analyzer with a nine channeltron detector. Survey spectra were acquired at a pass energy of 50 eV with the chamber operating at a pressure of 3 × 10^–10^ mbar. For analysis of the surface composition, core level intensities were normalized to their respective photoionization cross sections as calculated by Scofield^32^, as well as the instrument specific product of electron inelastic mean free path and analyzer transmission function determined by Speck^33^. SEM images were recorded by a Philips XL30ESEM-FEG purchased from ThermoFisher Scientific (https://www.fei.com/, Hillsboro, USA) with a standard setup: secondary electron detector, 3 keV acceleration voltage, and 6.5 mm working distance within a wide range of magnification (125–80,000).

## Results and discussion

### Electrochemical characterization

**Figure 2:**
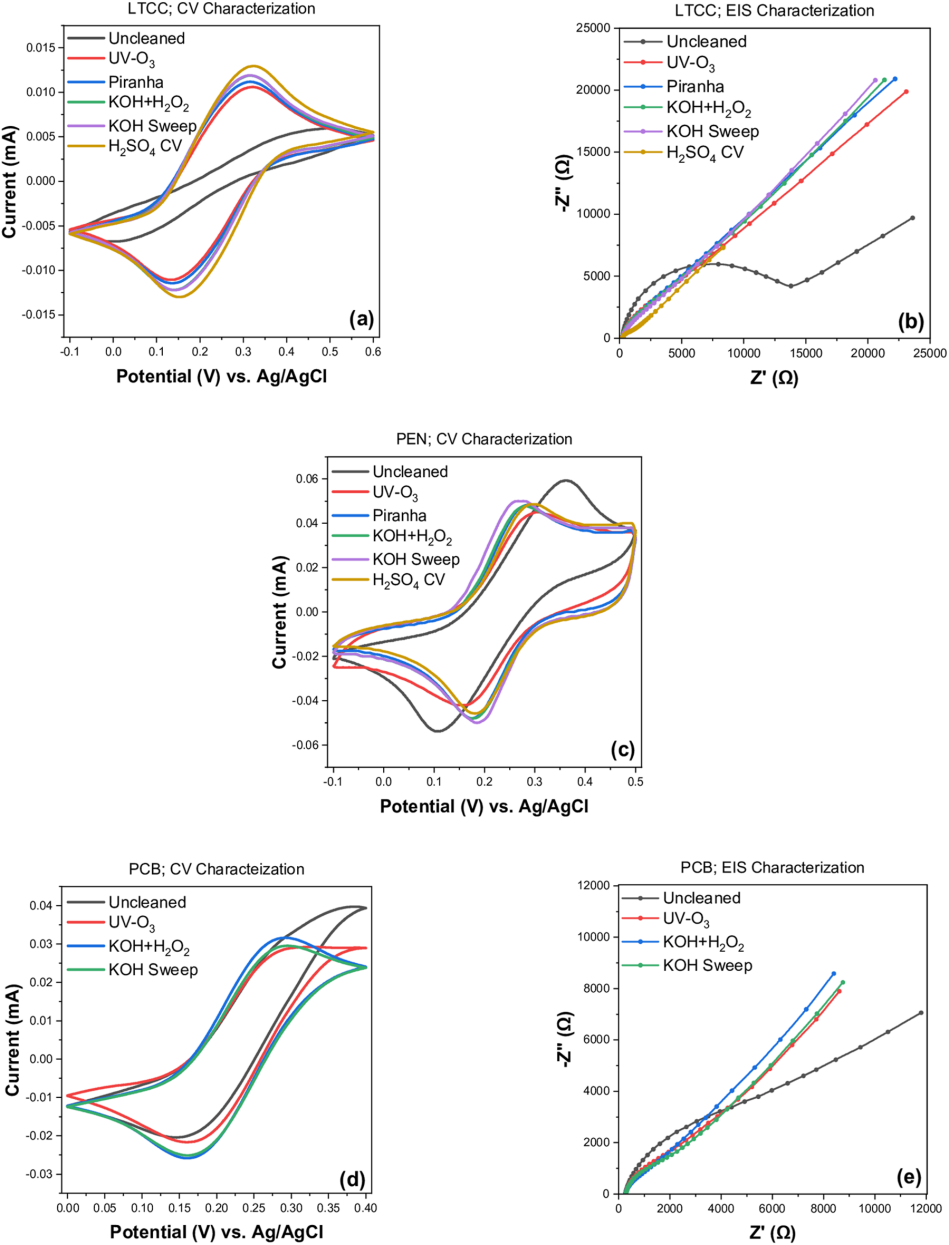
Electrochemical analysis of LTCC, PEN, and PCB sensor surfaces in PBS + 1 mM [Fe (CN)_6_]^3−/4−^ before and after each cleaning procedure. Cyclic voltammograms of (**a**) LTCC, (**c**) PEN, and (**d**) PCB sensors at 20 mV/s scan rate. Impedance curves of (**b**) LTCC and (**e**) PCB at amplitude of 10 mV within the frequency range of 0.1–100 kHz. KOH Sweep is the abbreviation of KOH + H_2_O_2_/KOH Sweep.

Cyclic voltammetry (CV) and electrochemical impedance spectroscopy (EIS) were employed to investigate the electroactivity of the gold surfaces before and after treatment with different cleaning procedures. Table 1 exhibits the potential difference between oxidation and reduction peaks (∆E_p_) and charge transfer resistance (R_ct_) values obtained from cyclic voltammogram and Nyquist plot of EIS for different Au sensors before and after cleaning procedures and rounded according to the rules of DIN 1333. The uncleaned LTCC sensors show the highest ∆E_p_ value (410 mV) without any specific oxidation and reduction peaks (Fig. 2a) and a wide semicircle diameter (Fig. 2b) corresponding to a very high resistance (1790 Ω cm^2^) that confirms almost full coverage of the surface with impurities. The considerable deviation from theoretical value of ∆E_p_ (58 mV at 25 °C) for single-electron transfer reactions such as in the [Fe (CN)_6_]^3−/4−^ couple on a perfectly clean gold surface^12,14^ can be due to the long-term storage in the laboratory environment and much more exposition to contaminants rather than other recently fabricated sensors. Accordingly, any changes on the electrode surface after each cleaning procedure strongly influence both the Peak-current potential-differences (∆E_p_) and charge transfer resistance (R_ct_) values. So that, ∆E_p_ from CV and R_ct_ from EIS can be used as a measure of surface cleanliness^12,14^. After UV-O_3_, the electrochemical values remarkably decreased to (104 mV, 233 Ω cm^2^) because of organic removal. Further decreases were subsequently found after different chemical and electrochemical treatments as summarized in Table 1.

**Table 1:** Peak potential difference (∆E_p_) and charge transfer resistance (R_ct_) values for different Au sensors before and after cleaning procedures.

Cleaning procedures	∆E_p_ (mV)			R_ct_ (Ω cm^2^)	
	LTCC	PEN	PCB	LTCC	PCB
Uncleaned	410 ± 80	271 ± 7	141 ± 13	1790 ± 270	670 ± 42
UV-O_3_	104 ± 6	104 ± 1	118 ± 4	233 ± 15	450 ± 40
Piranha	97 ± 1	93 ± 1	Corroded	170 ± 30	Corroded
KOH + H_2_O_2_	90 ± 2	94 ± 1	103 ± 1	90 ± 26	300 ± 10
KOH + H_2_O_2_/KOH sweep	89 ± 2	88 ± 0	110 ± 2	87 ± 11	365 ± 25
H_2_SO_4_ CV	83 ± 1	97 ± 1	Corroded	65 ± 8	Corroded

The R_ct_ values were normalized by the effective electrode area. Measurements are averaged over four separate samples.

Basically, for a reversible and fast electron transfer across the interface, the lowest ∆E_p_ and R_ct_ values will be expected, which is indicative of a clean and conductive surface area^12^. The lowest potential difference (83 mV) with sharp redox peaks and much lowest resistance (65 Ω.cm^2^) was achieved for LTCC cleaned with H_2_SO_4_ CV. In a perfect gold surface or bare gold electrode, the diameter of the semicircle portion in the Nyquist plot corresponding to the R_ct_ value is getting lost that only appeared as a straight line^31^. Figure 2b demonstrates a noticeable decrease in the diameter of the semicircle portion of the Nyquist plot after potential cycling in 0.5 M H_2_SO_4_, indicating noticeable removal of particles on the surface thereby reducing the charge transfer resistance of the LTCC sensors.

For PEN sensors before cleaning, a relatively high ∆E_p_ value of 271 mV could be evidently decreased with UV-O_3_ pretreatment (104 mV) and further improved following with other cleaning methods as seen in Fig. 2c. Eventually, the lowest peak separation was obtained after KOH + H_2_O_2_/KOH Sweep (88 mV) as shown in Table 1 and plotted in Fig. 2c indicating highly accelerated and facilitated charge transfer across the interface. Furthermore, the lowest standard deviation for PEN cleaned with combined (electro-) chemical alkaline procedure emphasizes the reproducibility of the PEN surfaces after treatment with this method. Interestingly, the same ∆E_p_ value (104 mV) for both LTCC and PEN after UV-O_3_ exposure could be achieved. While a little differences in ∆E_p_ value after chemical and electrochemical treatment confirms the fact that different kinds of Au electrodes are not affected by the cleaning chemicals to the same extent. A relatively low R_ct_ value (225 Ω cm^2^) for uncleaned PEN sensors indicated a highly conductive PEN surface even in the presence of impurities on it. Impedance measurements for all cleaned PEN sensors didn’t distinguish any semicircle portion in the EIS spectrum indicating a highly conductive PEN surface (diffusion-controlled)^31^. That’s the reason for missing the R_ct_ values of PEN electrodes in Table 1.

Electrochemical measurements for uncleaned PCB sensors result in (141 mV, 670 Ω cm^2^), not as much contaminated as LTCC. They were analyzed as soon as received before being too exposed to the laboratory environment. Further improvement in electrochemical response (118 mV, 450 Ω cm^2^) could be achieved with UV-O_3_ cleaning as clearly seen in Fig. 2d and e. In Piranha, uncertain evidence of corrosion was observed, while it severely intensified with electrochemical cycling in sulfuric acid. Therefore, corroded PCB chips in piranha and sulfuric acid were not characterized by electrochemical methods. Due to the oxidizing properties of H_2_O_2_ and to avoid formation of nickel-oxide on the PCB surface, the concentration of hydrogen peroxide was minimized to 50 mM and the cleaning solution was modified to 50 mM KOH and 50 mM 30% H_2_O_2_ in the ratio of 3:1 for this kind of sensor chips. After cleaning in KOH + H_2_O_2_, electrochemical values (103 mV, 300 Ω cm^2^) could be slightly improved and a well-distinguished pair of redox peaks appeared in the cyclic voltammogram of PCB sensors as seen in Fig. 2d. Nevertheless, after KOH Sweep an unexpected increase in ∆E_p_ and R_ct_ values was observed indicating oxide formation on the electrode surface following with a potential sweep. Although PCB surface could not be improved as much as LTCC and PEN, KOH + H_2_O_2_ is chosen as the most effective procedure to clean the PCB surface according to the electrochemical characterization results.

### Physicochemical characterization

**Figure 3:**
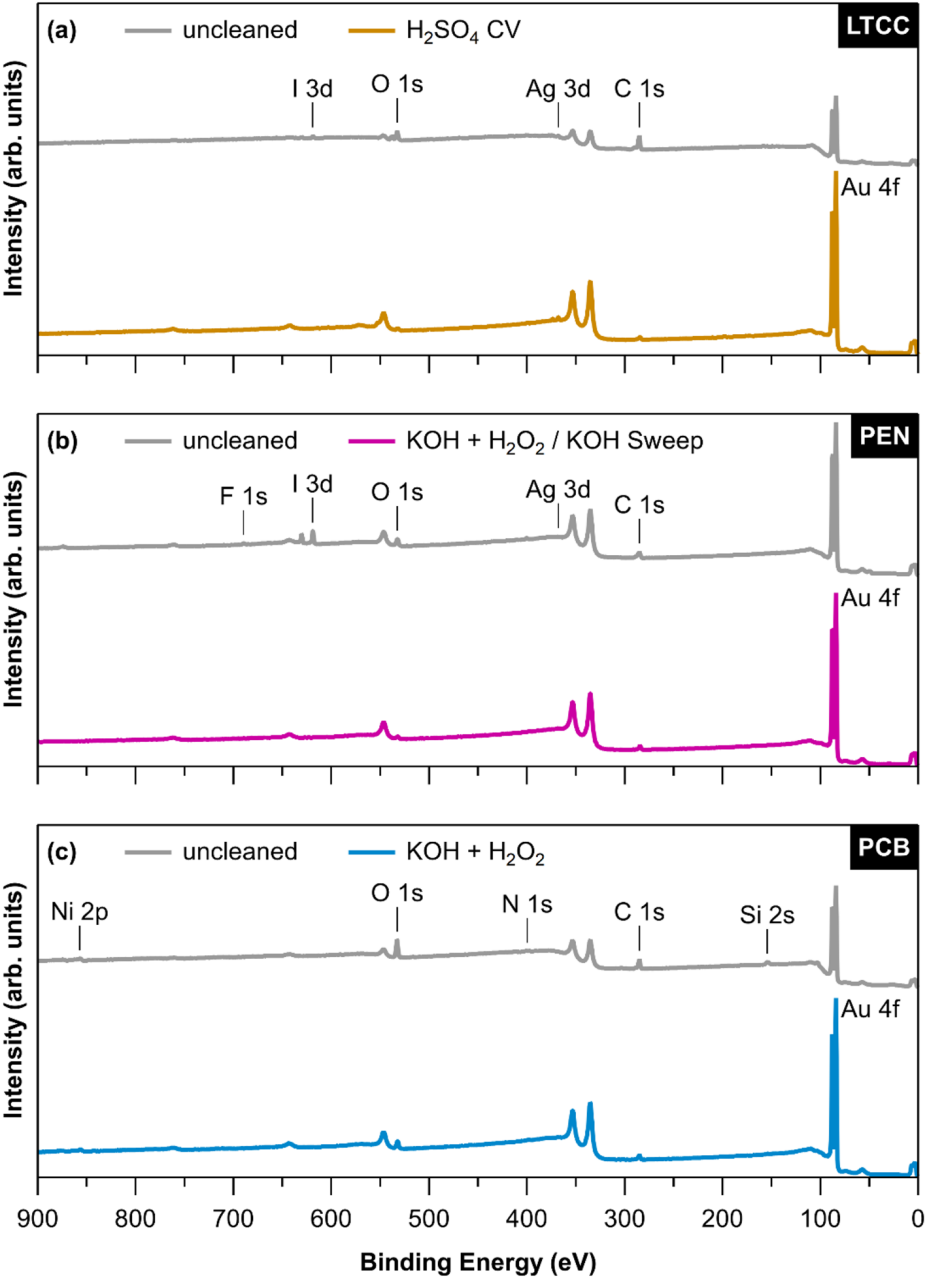
Representative XPS survey spectra of (**a**) LTCC, (**b**) PEN, and (**c**) PCB sensor surfaces. Spectra are shown for uncleaned samples and after the cleaning method deemed most efficient for each respective sample type and are offset for clarity. An increase in intensity of signals stemming from gold core level electrons can be observed, while contaminants such as carbon (C 1*s*) and oxygen (O 1*s*) are reduced.

X-ray photoelectron spectroscopy (XPS) and Scanning electron microscopy (SEM) provide useful information about the elemental composition and morphological properties of the surface. XPS survey spectra were recorded for each sample type before and after applying the different cleaning procedures, and the elemental composition was calculated from the intensity of the respective core level signals. Representative spectra of selected samples are shown in Fig. 3. The Au 4*f*, C 1*s*, and O 1*s* core levels were used to quantify the concentration of gold, carbon, and oxygen, respectively. LTCC and PEN samples additionally showed small amounts of iodine, silver, and fluorine, while signals from nickel and silicon were found on PCB samples. Since XPS is sensitive only to the topmost layers of the surface, the intensity of the gold core level signals increases upon removal of contaminants from the surface area. The results are summarized in Table 2 for all cleaning procedures.

**Table 2:** Summary of the XPS Analysis of surface composition of different sensor chips before and after cleaning procedures.

LTCC	Gold Au 4*f*	Carbon C 1*s*	Oxygen O 1*s*	Iodine I 3*d*_5/2_	Silver Ag 3*d*_5/2_	Other elements
Uncleaned	21.7 ± 5.3	61.7 ± 5.4	15.7 ± 0.1	0.4 ± 0.1	0.6 ± 0.2	-
UV-O_3_	60.4 ± 3.9	21.4 ± 1.5	15.7 ± 1.7	0.4 ± 0.2	2.2 ± 0.6	-
Piranha	70.1 ± 0.3	23.1 ± 1.1	6.3 ± 1.2	0.2 ± 0.1	0.5 ± 0.1	-
KOH + H_2_O_2_	64.4 ± 3.0	23.4 ± 1.3	8.6 ± 0.2	0.5 ± 0.3	3.2 ± 1.2	-
KOH + H_2_O_2_/KOH sweep	62.1 ± 4.0	20.2 ± 1.3	16.5 ± 7.3	-	1.3 ± 0.0	-
H_2_SO_4_ CV	70.1 ± 1.2	22.5 ± 1.7	5.9	-	1.6 ± 0.0	-

PEN	Gold Au 4*f*	Carbon C 1*s*	Oxygen O 1*s*	Iodide I 3*d*_5/2_	Silver Ag 3*d*_5/2_	Fluorine F 1*s*
Uncleaned	46.2 ± 5.9	34.5 ± 5.5	12.7 ± 1.6	4.9 ± 1.4	0.2 ± 0.0	1.7 ± 0.3
UV-O_3_	49.3 ± 1.0	31.1 ± 0.5	19.6 ± 0.6	-	0.1 ± 0.1	-
Piranha	54.8 ± 3.0	29.6 ± 1.4	11.4 ± 1.7	3.8 ± 2.3	-	0.5 ± 0.5
KOH + H_2_O_2_	62.4 ± 0.7	28.8 ± 0.5	8.6 ± 0.1	-	0.3 ± 0.1	-
KOH + H_2_O_2_/KOH Sweep	66.7 ± 1.4	25.7 ± 0.9	7.3 ± 0.4	-	0.3 ± 0.1	-
H_2_SO_4_ CV	57.9 ± 1.1	29.4 ± 0.9	12.6 ± 0.2	-	0.1 ± 0.1	-

PCB	Gold Au 4*f*	Carbon C 1*s*	Oxygen O 1*s*	Nickel Ni 2*p*_3/2_	Silicon Si 2*s*	Nitrogen N 1*s*
Uncleaned	20.9 ± 0.1	38.1 ± 2.1	26.4 ± 0.5	2.3 ± 0.1	10.1 ± 2.4	2.3 ± 0.5
UV-O_3_	43.3 ± 3.3	29.6 ± 2.6	19.5 ± 3.6	3.0 ± 1.8	1.5 ± 1.5	1.8 ± 0.6
Piranha	15.3 ± 4.9	33.7 ± 4.5	36.3 ± 0.9	11.9 ± 0.6	1.0 ± 0.9	-
KOH + H_2_O_2_	49.2 ± 2.4	27.8 ± 0.8	16.9 ± 0.9	3.3 ± 0.8	0.8 ± 0.8	2.1 ± 0.8
KOH + H_2_O_2_/KOH Sweep	46.2 ± 1.8	29.3 ± 0.8	20.1 ± 0.3	4.6 ± 0.8	-	2.1 ± 0.8

Elemental concentrations are given in percent. Measurements are averaged over multiple samples.

Quantitative XPS analysis for uncleaned LTCC chips (Fig. 3a) results in 21.7% gold, 61.7% carbon, and 15.7% oxygen. As XPS is a surface-sensitive technique, the removal of carbon contaminations to 21.4% leads to a huge increase in the intensity of the gold signals (60.4%) after UV-Ozone exposure. This confirms the effectiveness of this cleaning step to eliminate a considerable number of organic contaminants from the surface. Not much improvement in composition values can be seen for additional KOH + H_2_O_2_ or KOH + H_2_O_2_/KOH Sweep cleaning. The highest concentrations of 70.1% of gold could be achieved for LTCC sensors cleaned with potential cycling in 0.5 M H_2_SO_4_ and with Piranha treatment. Both techniques lead to comparable surfaces compositions with 22.5% (23.1%) carbon and 5.9% (6.3%) oxygen, respectively.

**Figure 4:**
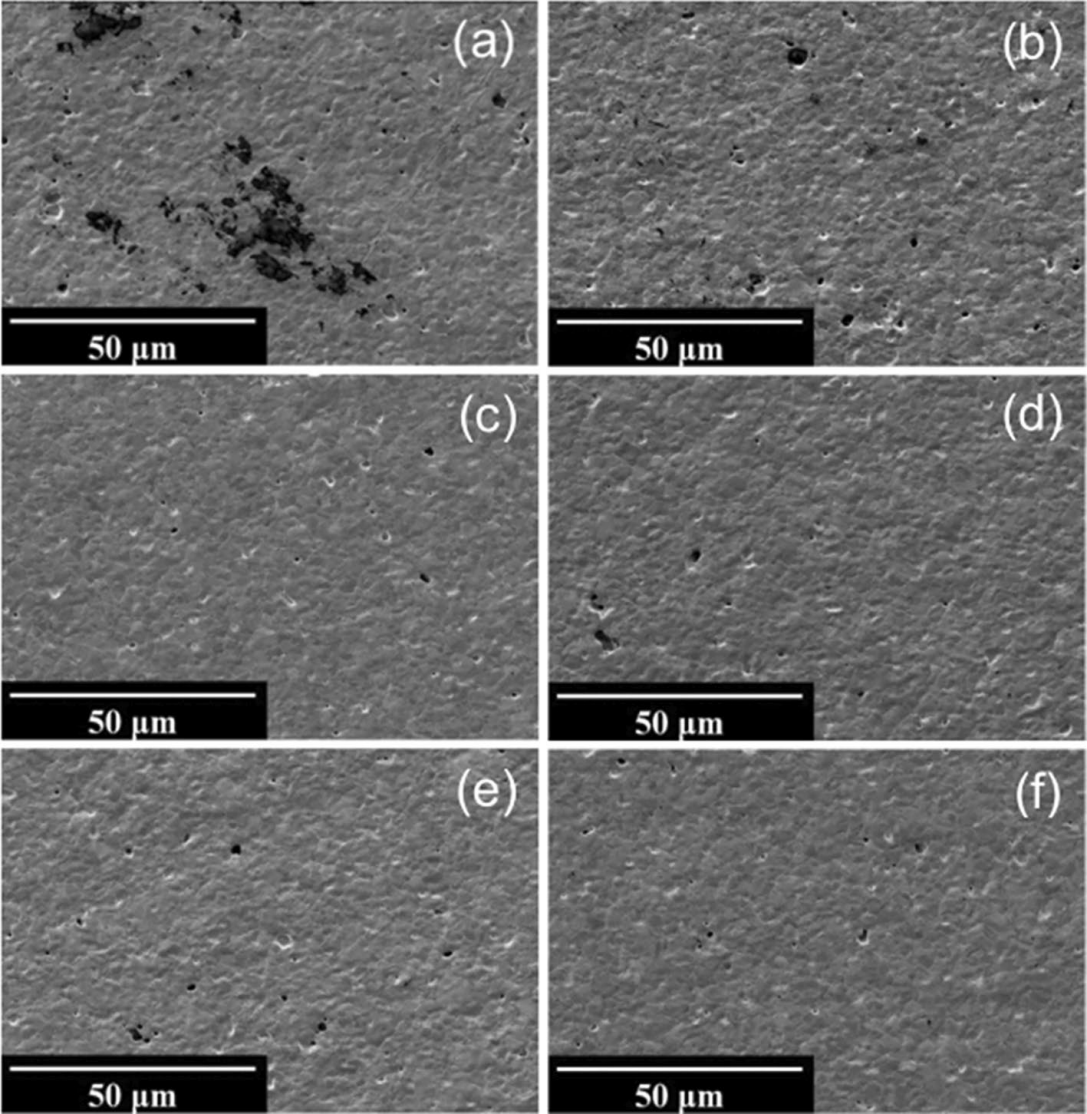
Scanning electron micrographs of LTCC surfaces (**a**) Uncleaned, (**b**) UV-O_3_, (**c**) Piranha, (**d**) KOH + H_2_O_2_, (**e**) KOH + H_2_O_2_/KOH Sweep, (**f**) H_2_SO_4_ CV; at 1000 × magnification.

SEM results of uncleaned LTCC surfaces (Fig. 4a) show a certain level of contaminants on the surface before cleaning which are significantly decreased during the procedure with UV-Ozone photoreactor (Fig. 4b). There are still a few numbers of dark spots on the surface correlated with either imperfections or residues of the contaminants which appeared to be removed with further (electro-) chemical treatments under alkaline and acidic conditions (Fig. 4c–f) and apparently be cleaned to the same extent. Although, Piranha resulted in the most uniform surfaces (Fig. 4c) with fewer particles and contaminants, the electrochemical characteristics are not as desirable as it seems to deteriorate the surface conductivity due to the insulating effect of non-conductive oxide layer formed on the surface of the sensor. Electrochemical cleaning in 0.5 M H_2_SO_4_ is chosen to be the most effective cleaning procedure for LTCC sensors, as it leaves the highest concentration of elemental gold and lowest content of carbon and oxygen on the surface in XPS measurements, while also showing the lowest peak potential difference and charge transfer resistance values in the electrochemical characterization.

For PEN sensors (Fig. 3b), already the uncleaned samples show less carbon and oxygen contaminations compared to the uncleaned LTCC sensors. Consequently, their gold concentration is relatively high at 46.2%, which can only be slightly improved via UV-O_3_ cleaning. However, UV-O_3_ cleaning removes unwanted elements such as iodine and fluorine. Due to the corrosive effect of Piranha solution, iodine and fluorine reappeared on the surface after Piranha treatment, while only slightly increasing the amount of elemental gold. The highest percentage of gold could be achieved with KOH + H_2_O_2_/KOH Sweep cleaning procedure, which shows excellent electrochemical characteristics and also confirms the results of^11,12^ for thin-film gold electrodes.

**Figure 5:**
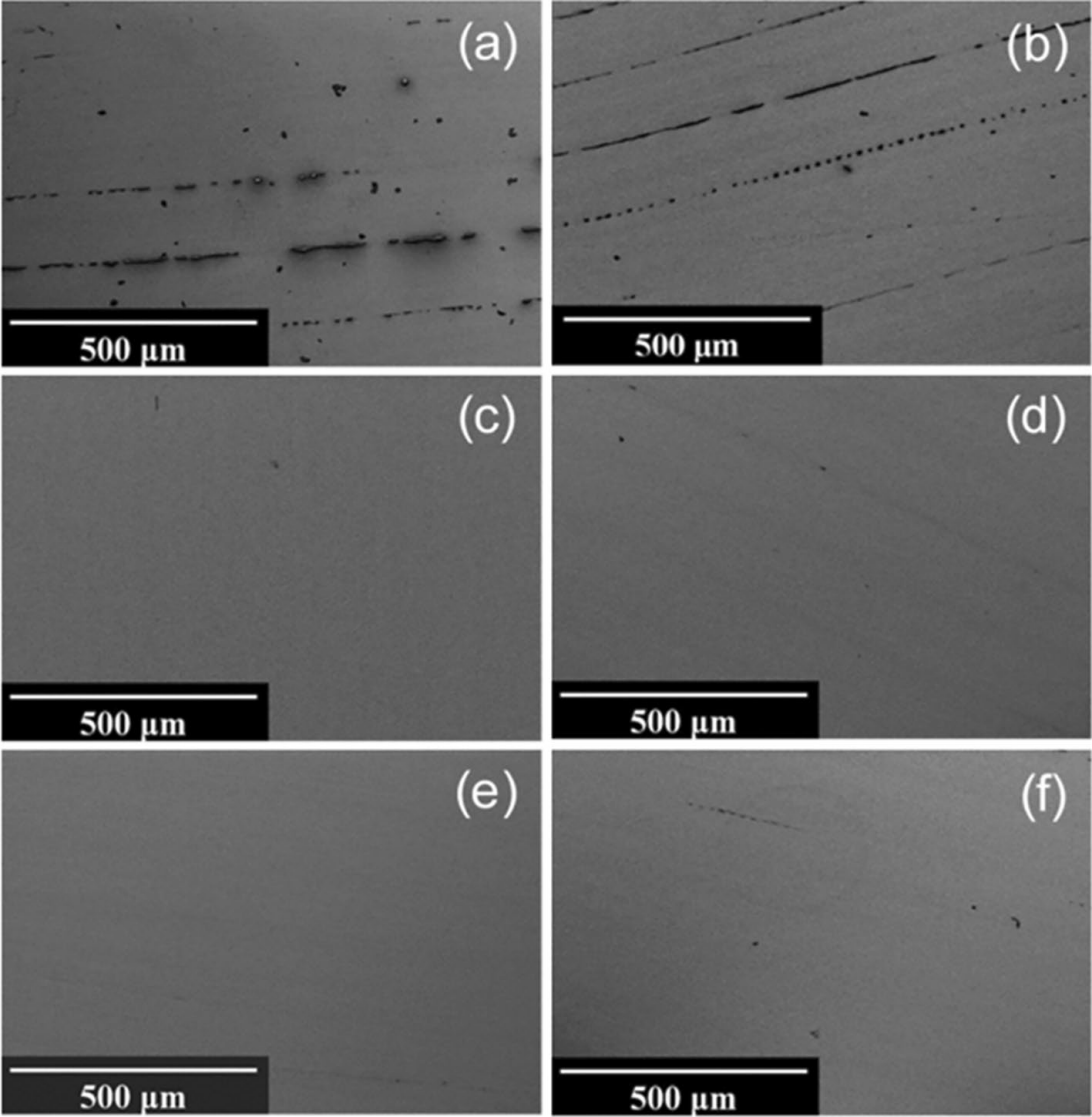
Scanning electron micrographs of PEN surfaces (**a**) Uncleaned, (**b**) UV-O_3_, (**c**) Piranha, (**d**) KOH + H_2_O_2_, (**e**) KOH + H_2_O_2_/KOH Sweep, (**f**) H_2_SO_4_ CV; at 125 × magnification.

Corrugated structure of inkjet-printed PEN surfaces results in a striped-like accumulation of contaminants on the surface of uncleaned PENs as shown in Fig. 5a. These are even visible to the naked eye which decreased with UV-O_3_ exposure (Fig. 5b) and relatively disappeared after subsequent surface treatments as shown in Fig. 5c–f. The most uniformity and surface improvement could be appeared in SEM images after cleaning with combined chemical and electrochemical alkaline treatments (Fig. 5e), indicating a significant removal of contaminants.

**Figure 6:**
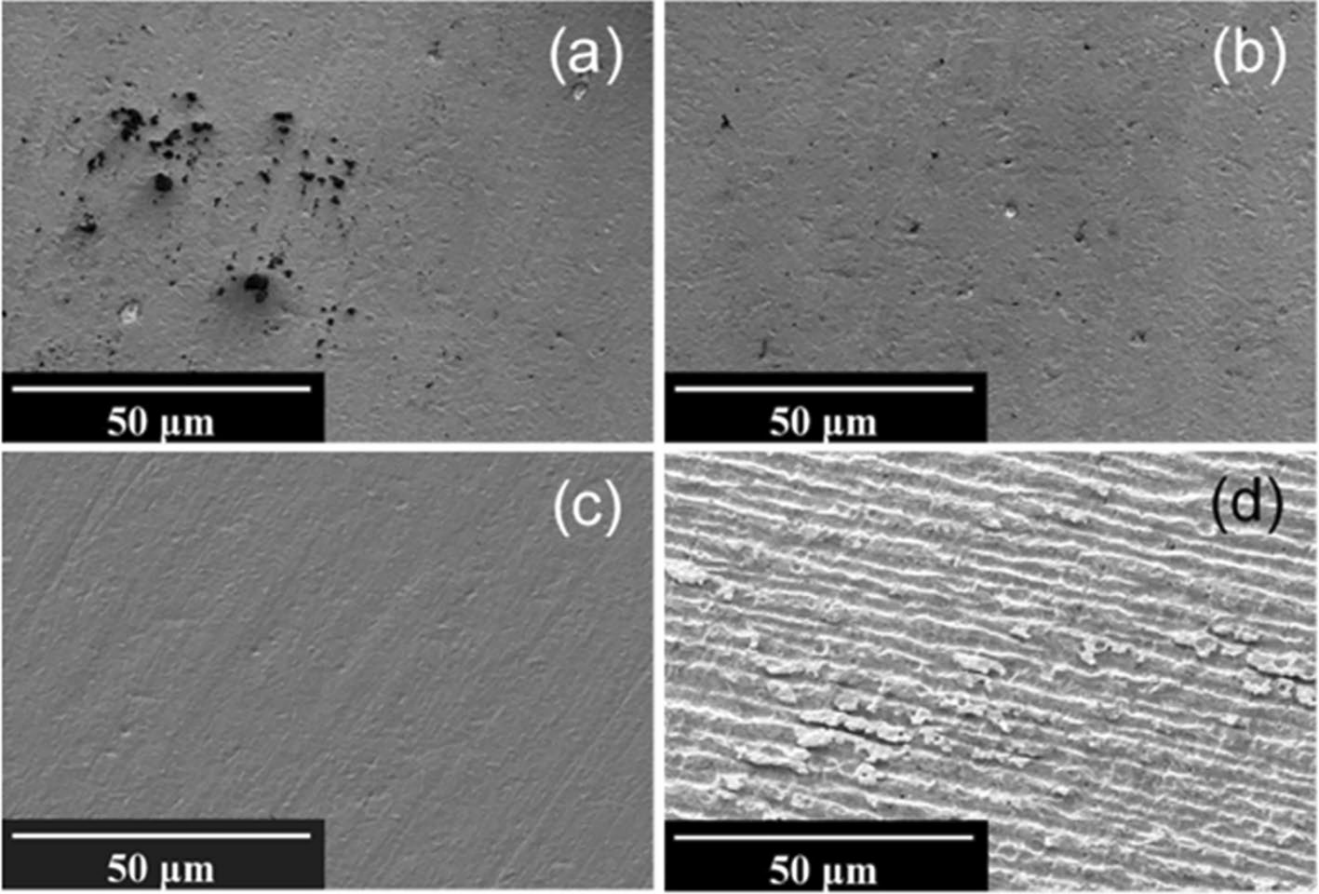
Scanning electron micrographs of PCB surfaces (**a**) Uncleaned, (**b**) UV-O_3_, (**c**) KOH + H_2_O_2_, (**d**) H_2_SO_4_ CV; at 1000 × magnification.

The surfaces of uncleaned PCB sensors (Fig. 3c) show a relatively high level of oxygen and carbon, while they also appear to be contaminated with silicon and nickel that could be either attributed to the significant impurities in the electroplating bath^34^ or likely leaked from the underlying layers. While UV-Ozone cleaning removes a considerable amount of the silicon, as well as some carbon and oxygen, still a high concentration of about 3.0% nickel remains on the surface, which can be oxidized in subsequent cleaning steps. Piranha treatment actually appears to further increase surface contamination with a higher percentage of oxygen and nickel as compared to UV-cleaned PCB samples. This could also be confirmed with XPS results which revealed a drastic increase in oxygen and nickel content after immersion in piranha which can be attributed to the possible nickel-oxide formation on the top of Au layer of PCB chips, while H_2_SO_4_ potential cycling causes severe corrosion leading to a morphology change as seen in the SEM analysis in Fig. 6d. KOH + H_2_O_2_ was found to be the most efficient cleaning method for PCB sensors, however the maximum concentration of gold on the surface achieved was still only around 50%, lacking behind the LTCC and PEN samples.

Significant differences in surface cleanliness and morphology of the PCB chips between the tested cleaning procedures were clearly illustrated in Fig. 6. A large number of organic contaminants on the surface before cleaning (Fig. 6a) were perfectly removed in UV-Ozone photoreactor as seen in Fig. 6b. While H_2_SO_4_ potential cycling caused severe corrosion and damage to the topography of the PCB (Fig. 6d), KOH + H_2_O_2_ could effectively remove the particles on the surface without changing the morphology of the gold surface (Fig. 6c).

The results obtained in the current study in light of previous works. In the current study, the cleaning procedures were explored on three different kinds of gold electrodes which were not reported elsewhere, while in previous works only one kind of electrode was explored. Findings by Fischer et al.^12^, on a thin-film gold electrode comparable with our PEN electrode in terms of gold thickness demonstrated that the combined (electro-) chemical alkaline pretreatment improves electrode cleanliness and increases gold content on a bare electrode like what we concluded as a most efficient cleaning procedure for PEN electrodes. The efficiency of the combined alkaline pretreatment was also confirmed by a recently published study^11^. In previous studies^26,35,36^, electrochemical treatment in 0.5 M H_2_SO_4_ was used to clean the screen printed gold electrodes what was found as a best cleaning method for LTCC screen printed electrode in this study. The XPS results in the current study shows a high percentage of elemental C and O on the uncleaned gold surface that is still a lot left even after cleaning procedures. The studies show that there are always a considerable amount of elemental C and O, even on the surface of Bulk gold^12^ that could be merely improved after surface cleaning^37^.

## Conclusion

In this study, the most effective cleaning procedure for Au electrodes fabricated with different materials and technologies was obtained. Potential cycling in sulfuric acid resulted in the highest gold content on LTCC surface, and the lowest ∆E_p_ and R_ct_ values which are known as a measure of surface cleanliness. Using a combined (electro-) chemical alkaline treatment known as KOH + H_2_O_2_/KOH Sweep in the results, the most desirable electrochemical and physicochemical properties of the PEN surface could be achieved. In general, a thick-film Au electrode like LTCC seems to be greatly clean with CV cycling in acid due to the removal of a thin gold layer during the electrochemical polishing, while in the case of PEN electrodes with only a thin layer of gold, a combined (electro-) chemical alkaline treatment seems to be the most effective method to clean the gold surface. Due to the presence of nickel under layer of ultra-thin Au layer of the electroplated electrodes like PCB and its substantial susceptibility to oxidation, harsh oxidizing cleaning methods like piranha or electrochemical techniques that sequentially oxidize and reduce the surface could not work. The most effective cleaning procedure for PCB was chemical cleaning in a low concentration of KOH + H_2_O_2_. Cleaning PEN, LTCC and PCB Au electrodes in piranha solution is not recommended due to its strong corrosive and oxidizing behavior resulting in the formation of metal oxide originating from the support material onto the gold surface which in turn significantly deteriorates the electrical conductivity. Importantly, the proposed cleaning procedures could be generalized to any kinds of Au electrodes depending on their gold thickness, substrates, and manufacturing technologies (Supplementary Information).

## Supplementary Information

Supplementary Information.

## Data Availability

The datasets generated during the current study are not publicly available due to the multiplicity of raw data and repetition of experiments but are available from the corresponding author on reasonable request.
