# Supplementary material for: Cleaning of LTCC, PEN, and PCB Au electrodes towards reliable electrochemical measurements
Source: Sci Rep. 2022 Nov 28;12:20431. doi: 10.1038/s41598-022-23395-3 (PMC9705539; doi:10.1038/s41598-022-23395-3)
Supplement: Supplementary file 1 — Supplementary Information. [file 41598_2022_23395_MOESM1_ESM.docx]

**Supplementary information (SI)**

**Table 1** Geometric information and effective surface area of LTCC, PEN, and PCB Au electrodes.

| Geometric parameters | Length  (mm) | Width  (mm) | Thickness  (µm) | A _geometric_  (cm^2^) | A _effective_  (cm^2^) |
| --- | --- | --- | --- | --- | --- |
| LTCC | 30 | 8 | 250 | 0.07 | 0.15 |
| PEN | 34 | 10 | 600 | 0.28 | 0.46 |
| PCB | 34 | 10 | 125 | 0.28 | 0.38 |


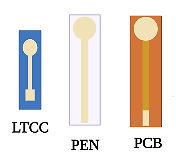


**Figure 1** Schematic representation of different Au electrodes.

**Table 2** EIS fitting parameters recorded at an amplitude of 10 mV on a DC potential of 0.2 V, within the frequency range of 0.1-100 kHz and fitted to the Randles equivalent circuit model. Measurements are averaged over four separate samples. KOH Sweep is the abbreviation of KOH + H_2_O_2_ / KOH Sweep.

| EIS fitting data | LTCC |  |  |  | PCB |  |  |  |
| --- | --- | --- | --- | --- | --- | --- | --- | --- |
|  | R_s_ (Ω) | R_ct_ (Ω) | C_dl_ ×10^-6^ (F) | Z_w_×10^-5^ (Ω/s ^1/2^) | R_s_ (Ω) | R_ct_ (Ω) | C_dl_×10^-6^ (F) | Z_w_×10^-5^  (Ω/s ^1/2^) |
| Uncleaned | 241±5 | 11900±1800 | 0.8±0.1 | 6.5±2.0 | 270±6 | 1760±110 | 3.9±0.1 | 10±1 |
| UV-O_3_ | 237±5 | 1550±100 | 2.4±0.2 | 4.2±0.1 | 282±6 | 1260±110 | 5.0±0.5 | 11±0 |
| Piranha | 227±4 | 1120±190 | 2.4±0.2 | 4.3±0.2 | - | - | - | - |
| KOH + H_2_O_2_ | 234±1 | 740±150 | 1.8±0.2 | 4.1±0.1 | 291±1 | 790±30 | 4.8±0.2 | 16±1 |
| KOH Sweep | 235±1 | 590±80 | 1.9±0.2 | 10.0±3.0 | 288±1 | 1025±36 | 5.0±1.0 | 10±1 |
| H_2_SO_4_ CV | 236±1 | 430±77 | 2.1±0.1 | 13.0±1.0 | - | - | - | - |


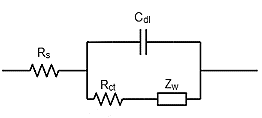


**Figure 2** Equivalent circuit model used for fitting of EIS data. R_s_: electrolyte resistance; C_dl_: interfacial capacitance; R_ct_: charge-transfer resistance, Z_w_: Warburg impedance.
